# Tapering of the cervical spinal canal in patients with distended or non-distended syringes secondary to Chiari malformation type I

**DOI:** 10.1186/1748-7161-10-S1-O52

**Published:** 2015-01-19

**Authors:** Zezhang Zhu, Shifu Sha, Xu Sun, Zhen Liu, Huang Yan, Weiguo Zhu, Jun Jiang, Yong Qiu

**Affiliations:** 1 Spine Surgery, Drum Tower Hospital of Nanjing University Medical School, China

**Keywords:** Chiari malformation, syringomyelia, scoliosis, cervical spinal canal, tapering

## Study design

A retrospective radiographic review.

## Objective

To investigate whether taper ratio of the cervical spinal canal differs between distended and non-distended syringes in patients with Chiari malformation-associated syringomyelia.

## Methods and materials

Seventy-seven patients were divided into two groups based on the syrinx/cord (S/C) ratio: one with distended syrinx (S/C ratio > 0.5) and the other with non-distended syrinx (S/C ratio < 0.5). Anteroposterior diameter of the spinal canal was measured at each cervical level on midsagittal T2-weighted MR images, and a linear trend line was ft by least-square regression to calculate the taper ratio. With the Kruskal-Wallis test, taper ratios were compared between the two groups and further evaluated with respect to age and gender.

## Results

Of the 77 patients, 44 were classified as having distended syrinx (group D), and 33 were assigned into the non-distended group (group ND). Age, gender, syrinx location and the extent of tonsillar ectopia were comparable between the two groups (P > .05). In group ND, the taper ratios for C1-C7, C1-C4 and C4-C7 averaged -0.73 ± 0.56, -1.61 ± 0.98 and -0.04 ± 0.54, respectively, all of which were significantly steeper than those observed in group D (P <.001, .001 and .031, respectively). Regarding the average diameters plotted by cervical level, the narrowest region of the canal was found to occur at C4 in both groups. Additionally, no significant differences in taper ratio were noted between males and females, or between older < 14 years) and younger patients (> 14 years).

## Conclusions

Morphology of the cervical spinal canal was found to be different between patients with distended and non-distended syringes, indicating a reciprocal interaction between the syrinx and the cervical spine anatomy. The integral role of spinal canal dimensions in the pathogenesis of syringomyelia, however, has yet to be explored by longitudinal studies involving both CMI patients and normal controls.

